# Iron deficiency and iron treatment in the fetal developing brain – a pilot study introducing an experimental rat model

**DOI:** 10.1186/s12978-018-0537-0

**Published:** 2018-06-22

**Authors:** Torben Moos, Tina Skjørringe, Lars Lykke Thomsen

**Affiliations:** Laboratory of Neurobiology, Biomedicine Group, Department of Health Science and Technology, Aalborg University, Fr. Bajers Vej 3B, 1.216, DK-9220 Aalborg East, Denmark

**Keywords:** Brain, Development, Fetal, Iron, Pregnancy

## Abstract

**Background:**

Iron deficiency is especially common in women during the reproductive age and it is estimated that 52% of pregnant women have iron deficiency anemia. Maternal iron deficiency with or without anemia in pregnancy may have consequences for the fetus, where it may have an impact on the cerebral development of the brain. Both animals and adult human studies support that iron deficiency affects psychomotor development, behavioral traits, and cognitive functions in the offspring. However, it has not yet been established whether the availability of sufficient iron is particularly important in certain phases during brain development, and whether possible damages are reversible if iron supplementation is provided during pregnancy. Here we report results from a pilot study in an experimental rat model suitable for introducing iron deficiency in the fetal rat brain.

**Methods:**

The model was utilized for examination of the potential to reverse changes in fetal brain iron by maternal parenteral iron administration. Fertilized females subjected to iron deficiency without anemia were subcutaneously injected with iron isomaltoside at the day of mating (E0), 14 days into pregnancy (E14), or at the day of birth (Postnatal (P) 0). Blood, brain and liver in the offspring were examined on P0 or in adulthood on postnatal day P70.

**Results:**

Maternal iron restriction during pregnancy led to significantly lower levels of iron in the brains of newborn rats compared to levels in pups of iron sufficient mothers. Females fed ID diet (5.2 mg/kg Fe) had offspring with significantly lower cerebral iron compared to a control group fed a standard diet (158 mg/kg Fe). Injection of IIM to pregnant ID females on E0 or E14 yielded normalization of Fe in the developing brain known to express elevated levels of capillary transferrin receptors, indicating that the administered iron passed the placenta and fetal blood brain barrier.

**Conclusions:**

In future studies, this translational model may be applied to examine morphological and biochemical consequences of iron deficiency and iron deficiency treatment in the developing fetal brain.

## Background

According to the World Health Organization (WHO), iron deficiency affects approximately 20% of the population worldwide equal to approximately 1.4 billion people. Iron deficiency is especially common in women during the reproductive age and in young children [1]. A major consequence of iron deficiency is anemia, and in developing countries, it is estimated that 52% of pregnant women have iron deficiency anemia [1]. The frequencies of iron deficiency anemia in the UK in the first, second, and third trimesters are approximately 2, 8, and 27%, respectively [2].

Maternal iron deficiency anemia in pregnancy may have consequences for both mother [3] and fetus/newborn. Some consequences for the fetus include ‘small for gestational age’ (SGA) and intrauterine growth restriction (IUGR), which can complicate the neonatal period and by itself affect brain development of the fetus/newborn [4–6]. Iron is an essential co-factor for many enzymes, which are important in normal cell physiology, e.g. mitosis. Thus, there is a good reason to believe that iron deficiency in the developing brain even without fetal anemia may adversely affect the developing fetal brain [7]. In the central nervous system, iron is a co-factor for a variety of proteins and lipids vital for the normal cellular function. Iron is essential for cell division [8], including neuronal precursors of the developing brain, hence making gestational iron deficiency a serious challenge.

Iron deficiency in experimental animals affects dendritic growth, synaptic formation, and the function of oligodendrocytes, i.e. axon myelination [7, 9, 10]. Iron deficiency affects specific regions of the developing brain, including basal ganglia and hippocampus, which are involved in recognition memory, cognitive functions, and other higher cerebral functions [4, 11, 12]. Both experimental animals and adult human studies support the notion that iron deficiency affects psychomotor development, behavioral traits (anger, fear, anxiety), and cognitive functions in the offspring [7, 13–23]. However, it has not yet been established whether the availability of sufficient iron is particularly important in certain phases during brain development, and whether possible damages are reversible if maternal iron supplementation during pregnancy is applied. Further, little is known about transfer of iron across the placenta and the fetal blood-brain barrier. Properly designed interventional trials of iron treatment controlling for cofactors, correct dosing, and way of administration etc. either as maternal treatment during pregnancy or in the neonates are still warranted. Animal models of iron deficiency treatment may provide some information of how iron deficiency effects morphological brain development and translational animal models are relevant in this aspect as it allows controlled experimental conditions and postmortem tissue for biochemical and morphological examinations. We here describe results from a pilot study in an experimental rat model suitable for introducing iron deficiency in the developing brain.

This model was utilized for examination of the potential to reverse eventual changes in fetal brain iron by means of maternal parenteral (i.e. subcutaneous) iron administration.

## Methods

Eight female rats aged postnatal day 42 (P42) were fed a normal diet with an iron content of 158 mg/kg (Altromin, Germany) (control rats (group N)) for six + six weeks, equal to 12 weeks. In parallel, 56 female rats were also initially fed the normal diet for six weeks until they reached P42. Then they were given an iron deficient diet for another six weeks with an iron content of 5.2 mg/kg (Altromin, Germany). The rats were fertilized by male rats fed the normal diet and maintained on the two different diets. For iron supplementation, the maternal rats fed the diet with a low iron content were injected subcutaneously with iron isomaltoside (Monofer®, Pharmacosmos A/S, Holbaek, Denmark) at a dose of 80 mg/kg on day E0 (the day of conception, group A), E14 (14 days after conception, group B), or the day of birth, day P0 (group C). Twelve pups aged P0 (date of birth) were obtained from the pregnant rats of groups A, B, C, and N (controls), euthanized and examined for brain iron levels. The Danish Experimental Animal Inspectorate under the Ministry of Food and Agriculture (permission no. 2013–15–2934 − 00776) approved the handling of the animals in this study.

The brain stem of the pups were dissected and used for detection of the concentration of iron by Inductively Coupled Argon Plasma with Optical Emission Spectrometry detection (ICP-OES) (ICAP 6300 Duo View, Thermo Scientific). In brief, the tissue samples were freeze dried, homogenized, and transferred to Teflon vessels. The samples were then digested using microwave assisted acid digestion with 8 mL concentrated nitric acid for 10 min at 1200 W. Four cooled digestates were diluted to volume with ultrapure water and transferred to plastic flasks, and allowed to settle before being analyzed for iron. Iron concentration was determined by ICP-OES. All were measured axially at two to three wavelengths. All data are shown as mean ± standard error of the mean (SEM). One-way Analysis of Variance (ANOVA) analysis with Tukey’s multiple comparison post-hoc tests was performed using the GraphPad Prism version 6.01 software in order to compare data from all groups to controls or to the respective dietary group (e.g. A vs B). Values of *p* < 0.05 were considered statistically significant.

## Results

**Fig. 1:**
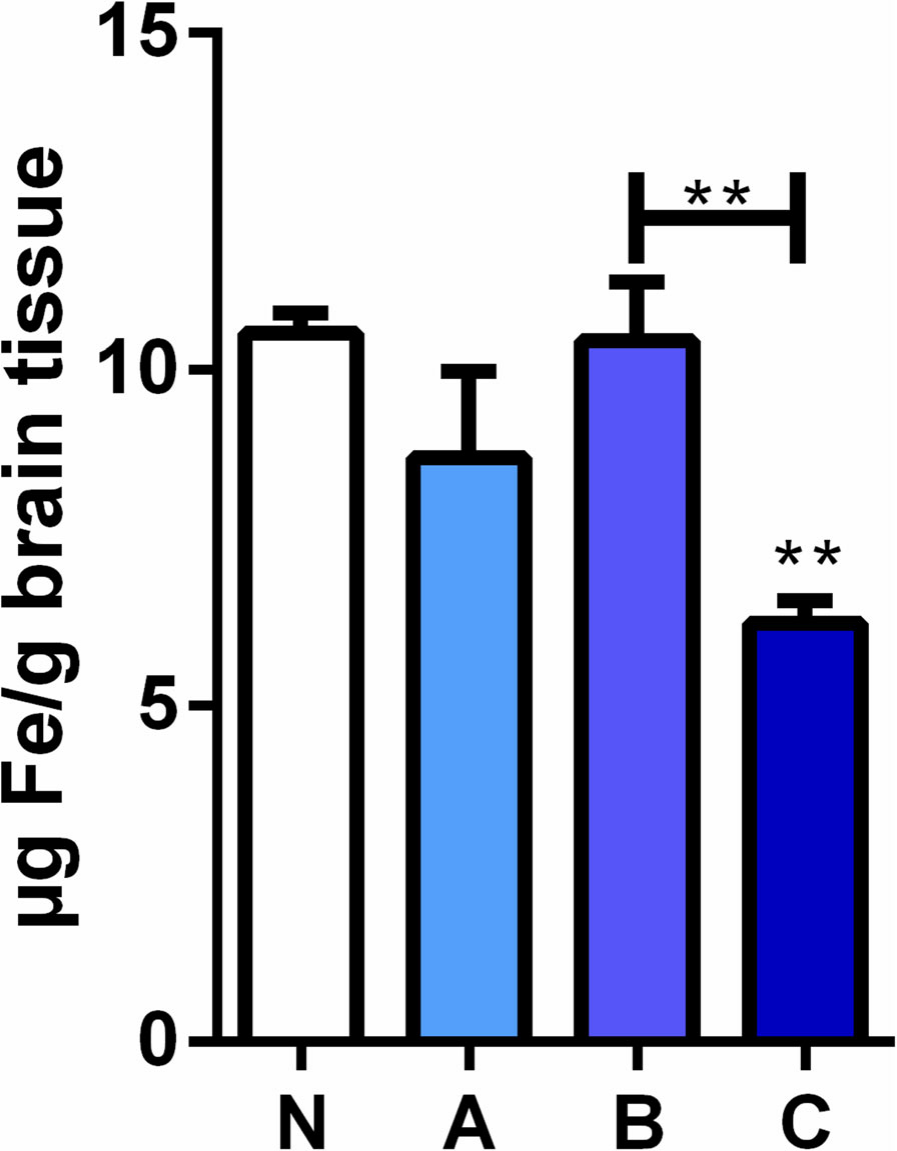
The levels of iron in brains of newborn rat pups of mothers fed with iron deficient diet with or without supplementation. In the brain iron (Fe) is significantly lower in the pups from mothers on the iron deficient diet (C group) compared to the pups from mothers on the iron deficient diet treated with parental iron isomaltoside at E 14 (B group) and pups from normal fed mothers equal to an iron sufficient diet (N group). The A group which represents pups from mothers on the iron deficient diet treated with parental iron isomaltoside at E0 did not differ from either of the B or N groups, but it was still higher than in C. Data are presented as mean ± SEM (*n* = 4–5). * *p* < 0.01

The pups of iron deficient mothers that did not receive parenteral iron during pregnancy (C) had a significant, 40% reduction (6.2 ± 0.3 μg/g) of the iron level in the brain compared to pups of the control mothers (N, 10.5 ± 0.3 μg/g) (Fig. 1). In contrast, pups of iron deficient mothers that received parenteral iron two thirds into the pregnancy (E14, B group) had amounts of brain iron levels that did not differ from the control group at birth (Fig. 1). Pups of mothers treated with iron at the day of conception (E0, A) had raised concentrations of iron compared to the group that did not receive iron (Fig. 1).

## Discussion

We have in this brief communication described a rat model of iron deficiency in which maternal iron restriction during pregnancy led to significantly lower levels of iron in the brains of newborn rats compared to levels in pups of iron sufficient fed mothers. Parenteral administration with iron isomaltoside to the pregnant female rats with iron deficiency restored normal levels of iron in the brain of the newborn pups. Hence, the administered iron passed the placenta and fetal blood brain barrier. The parental supply with iron isomaltoside also restored hemoglobin level in the developing rat, as at the day of birth the newborn rats of ID mothers (group C) had a significantly lower hemoglobin level (3.6 ± 0.18 mmol/L) than the newborn rats of mothers fed a normal diet (6.02 ± 0.09 mmol/L) (Moos et al., in preparation).

In future studies, the translational model of the present brief study may be applied to examine morphological and biochemical consequences of iron deficiency and iron deficiency treatment in the developing fetal human brain if confirmed in extended and repeat studies. Although this will be difficult to perform in humans and thereby a limitation in the translation of the present study, it should nonetheless be conceivable for clinical research by administering iron to human mothers early and later in pregnancy. Thereby, it should be possible to measure serum concentrations of iron in neonates to follow the cognitive and behavioral outcome of children over years.

## Conclusions

This translational study may provide useful data allowing for optimal planning and transition into clinical trials as the translational model may be extended to also include assessment of associated behavioral consequences and potential correlations to morphological and biochemical consequences of iron deficiency and iron deficiency treatment in the developing fetal brain. The model also represents a unique model making it possible to examine details regarding regulation of maternally administered iron across the placenta as well as the fetal blood-brain barrier. Further, it allows future studies of optimal timing and dose levels of iron treatment in the pregnant mother to optimize fetal brain development in iron deficient mothers.
